# A Population-based Screening of Type 2 Diabetes in High-risk Population of Yasuj, Iran

**Published:** 2014-12

**Authors:** Mitra Safari, Behrouz Yazdanpanah, Behzad Yazdanpanah, Ali Mobasheri

**Affiliations:** ^1^Yasuj University of Medical Sciences, Yasuj, Iran; ^2^Medical Student, Shiraz University of Medical Sciences, Shiraz, Iran; ^3^Yasuj Education Office, Yasuj, Iran

**Keywords:** Diabetes, Population-based studies, Risk factors, Screening, Iran

## Abstract

Complications associated with diabetes can be prevented by early diagnostics. A high-risk population was screened for diabetes, and the prevalence of undiagnosed diabetes mellitus (DM) and impaired fasting glucose (IFG) were used for examining the impacts of lifestyle, social and anthropometric features, and other risk factors. The target population comprised 30-65 years old residents from the western suburbs of Yasuj. Homes were approached, and a standard questionnaire was used for collecting information on sex, blood pressure, weight, height, and BMI for each participant. The high-risk participants were recognized according to the National Diabetes Prevention and Control Committee criteria and were introduced to an assigned laboratory. Blood samples were collected after 12-hour fasting for the measurement of total cholesterol, triglycerides and fasting glucose levels. The statistical analysis was performed with the SPSS statistical package, using a logistic regression model. Out of 2,569 individuals, 1,336 (52%) were with high-risk diabetes, 71.5% were female, and 28.5% were male. Of 191 (7.4%) individuals with known diabetes, 5 (2.6%) had type 1 diabetes; 881 (66.9%) out of 1,336 high-risk individuals were referred to assigned laboratory. Of 881 high-risk individuals, 157 (17.8%) had fasting blood sugar (FBS) ≥126 mg/dL and 118 (13.4%) had FBS between110 and 125 mg/dL. Percentages of participants with triglyceride ≥150 mg/dL and cholesterol ≥200 mg/dL were 298 (33.8%) and 207 (23.5%) respectively. Diabetes was associated with ageing, dyslipidaemia, family history of diabetes, lower physical activity on occupation, intake of lower dietary fibre, and non-literacy in the sampled population. This study suggests that diabetes is a common health problem in this area. Furthermore, considerable rate of newly-diagnosed diabetes signifies the importance of the screening programme.

## INTRODUCTION

The prevalence of diabetes has increased during the last two decades in countries with low and middle income (1). This trend, which is almost completely due to type 2 diabetes, is expected to rise (2). The rise in the prevalence of type 2 diabetes will increase the likelihood of patients at risk of serious diabetes-related complications. Type 2 diabetes increases the risk of myocardial infarction two times and the risk of having a stroke two to four times. Furthermore, type 2 diabetes is one of the leading causes of blindness, limb amputation, and kidney failure (3-4).

The 2010 global diabetes prevalence among adults aged 20-79 years is estimated at 6.4%, affecting 285 million adults. Between 2010 and 2030, the adults with diabetes are expected to rise by 70% and 20% in developing and developed countries respectively (5). Environmental and lifestyle factors are among the main causes of the dramatic increase in the prevalence of type 2 diabetes (6-7).

The associations between body mass index (BMI), lipids, hypertension, smoking, physical inactivity, low education, dietary patterns, family history and specific genes with type 2 diabetes have been documented (8-11). The Middle East is expected to bear the highest increases in the absolute burden of diabetes in the coming decades. This increase is anticipated to affect the economically-productive 45 to 64 years old individuals (12).

The diabetes mellitus (DM) is the main cause of a common disease with increasing incidence and a variable geographical prevalence in Iran. A recent study reported 9.8% prevalence in the highly-urbanized capital of Iran, Tehran (13). The prevalence of DM in the Isfahan Healthy Heart Program was reported at 6.7% and 5.3% in urban and rural areas and 5.4% and 7.1% in males and females respectively (14). Crude prevalence of diabetes and age-adjusted prevalence were reported at 13.4% and 11% respectively in Booshehr while prevalence at Yazd was reported at 16.3% (15).

The prevalence of diabetes in Iran is estimated at 7.7% for adults aged 25-64 years, affecting 2 million individuals, where only one-half are undiagnosed. Furthermore, an additional 16.8% or 4.4 million of adults have been reported to have impaired fasting glucose (IFG) (16). The prevalence of type 2 diabetes by systematic review between 1996 and 2004 in those aged >40 years has been estimated at 24% in Iran and increases by 0.4% with each year after 20 years of age (17). The complications associated with diabetes can be prevented by early diagnosis, intense monitoring, and proper treatment. Diabetes is a major concern, and both diabetes and public health organizations worldwide have expressed the need for screening in asymptomatic individuals (18,19).

No representative population-based study has been undertaken to estimate the prevalence of diabetes and risk factors in the Kohgiloyeh and Boyerahmad province located in southwestern Iran. This study was carried out to estimate the prevalence of the undiagnosed DM and IFG in high-risk population and to examine their relationship with lifestyle, social and anthropometric features, and other risk factors.

This community-based study was approved by the Technology and Research Council of Kohgiloyeh and Boyerahmad province and covered the period of 2009-2010.

## MATERIALS AND METHODS

### Study subjects

The target population comprised 30-65 years old residents of the western suburbs of Yasuj. All individuals were screened in the survey, and those at high risk were included in the study.

### Field survey

In total, 86 team members, mostly volunteer medical university students were involved in the field work. At least 2 days before the study, team members attended a training course covering specific local arrangements, completion of the questionnaire, anthropometric, physical, and blood pressure measurements. Teams were supervised by healthcare providers. A core mobile team was tasked for standardization and quality control between the teams. During the field survey, instruments had been checked every morning. All homes were approached. After participants’ consent, a standard questionnaire, which was approved by the National Diabetes Prevention and Control Committee, covering social and demographic characteristics, socioeconomic status, education, medical history, lifestyle, food intake, and reproductive history (in women), was administered (20).

Body-weight and height were measured while subjects were wearing light clothing without shoes, and BMI was calculated. Height was measured to the nearest cm, using a tape stuck to the wall with the subject standing erect. Weight was measured to the nearest 0.1 kg, using a digital bathroom scale. All the bathroom scales were calibrated daily, using a two-kg counterweight. Blood pressure was measured twice in a 5-minute interval, in sitting position, after 10-minute rest, and the mean was taken in all cases. Both systolic (SBP) and diastolic blood pressure (DBP) were recorded at the level of appearance and disappearance of sound respectively. A combination of leisure, home and occupation-related activities was considered as physical activity. The leisure-related activity was given a score of 1 for hobbies that involved no physical activity, 2 for hobbies that involved physical activity or active sport 1 to 2 day(s) per week, 3 for active sport ≥3 days per week. The home and occupation-related activities were given a score of 1 for unemployment and work involving no physical activity, 2 for work that involved physical activity, and 3 for heavy work. Physical activity was classified as ‘sedentary’ if the sum of both types of activity was equal to 2, ‘moderate’ if the sum was 3-4, and ‘heavy’ if the sum was 5-6.

Current smokers represented subjects smoking at least one cigarette a day. Consumption of vegetables and fruits was measured as number of days of consumption in the past week. Consumption of saturated oil was based on the usual use of saturated oil in cooking.

The high-risk participants were recognized using the National Diabetes Prevention and Control Committee criteria (20) which consider individuals with history of diabetes in the first-degree relatives, blood pressure ≥140/90 mmHg, body mass index ≥30 kg/m^2^, and women with history of stillbirth, abortion ≥2, gestational diabetes, and giving birth to a baby weighing >4 kg as high-risk individuals.

### Laboratory tests

High-risk people were introduced to an assigned laboratory. Blood samples were drawn after 12-hour fasting for the measurement of total cholesterol, triglycerides and fasting glucose levels. Levels of plasma glucose, total cholesterol, and triglycerides were determined by the enzymatic GOD, PAP-CHOD, and GPO-PAP methods, using the Main dray-B 2000 autoanalyzer respectively. Known diabetes mellitus (KDM) were defined when the subject reported a history of physician or healthcare professional diagnosing diabetes, taking oral hypoglycaemic tablets or insulin injections, and newly-diagnosed diabetes was identified based on WHO criteria (21) as fasting blood sugar (FBS) ≥126 mg/dL for two times of testing compared to those without KDM having fasting blood sugar of ≥110 mg/dL but <126 mg/dL was designated as having impaired fasting glucose (IFG). Individuals with FBS <110 mg/dL were defined as having normal glucose tolerance (NGT). Cutoff values for serum lipids were ≥200 mg/dL cholesterol and ≥150 mg/dL triglyceride (10,11). Newly-diagnosed people were referred to physician for consultation and received standard medical care.

### Statistical analysis

Statistical analysis was performed with the SPSS package (version 17). The baseline values are reported as numbers (proportions) for categorical and mean±SD for the continuous variables. For normally-distributed quantitative variables, group comparisons were done using independent samples *t*-test or by ANOVA. Relationship among different groups and qualitative variables were analyzed using chi-square or Fisher's exact test where appropriate. Variables found to be associated with IFG and DM in univariate analysis were included in a multinomial logistic regression model. The dependent variable had three categories based on participant's fasting plasma glucose: normal glucose tolerance (NGT), impaired fasting glucose (IFG), and diabetes mellitus (DM). The independent variables used in the analysis were age, total cholesterol (TC), triglyceride (TG), consumption of vegetables and fruits, physical activity on occupation, educational level, and a family history of diabetes. A p value of less than 0.05 was considered significant.

## RESULTS

Out of 1,320 households, 24.4% could not be approached, 49 (3.7%) refused participation, and 57 (4.3%) had no individuals aged 30-65 years. A total of 2,639 people were examined at field survey. Seventy cases were excluded due to flaws. Out of 2,569 individuals, 43.6% were male and 56.4% female. General characteristics of the participants who took part in the survey are presented in Table 1. Proportion of women with elementary schooling and university education were 39.7% and 5% respectively. Corresponding figures for men were 15.6% and 35.7%.

Based on the National Diabetes Prevention and Control Committee criteria, 1,336 (52%) individuals were at high risk of diabetes, with a gender ratio of 71.5% female and 28.5% male (Table 2). The most frequent criteria in the high-risk individuals were obesity 927 (36.1%), followed by high diastolic blood pressure 623 (24.3%). High systolic blood pressure was more frequent in women than men (14.4% vs 10.3%).

General characteristics and lifestyle variables in participants are presented in Table 3. Among male participants, 382 of 1,119 (34.1%) were classified as high-risk compared to females, with 954 of 1,450 (65.8%) at high risk. Significant differences were observed between genders and age-groups, educational level, physical activity, smoking, and consumption of saturated oil. Out of 191 (7.4%) individuals with known history of diabetes, 5 (2.6%) had type I diabetes. Of the 1,336 high-risk individuals, 881 (66.9%) were referred to assigned laboratory for blood sampling. Out of 881 individuals who were assigned to laboratories, 157 (17.8%) had fasting blood sugar (FBS) ≥126 mg/dL, 118 (13.4%) with FBS between 110 and 125 mg/dL; 298 (33.8%) with triglyceride ≥150 mg/dL and 207 (23.5%) with cholesterol ≥200 mg/dL.

Statistically significant demographic and biomedical indicators, affecting dependent variable classified as NGT, IFG, and DM in high-risk population, are presented in Table 4. Age-groups, triglyceride, total cholesterol, physical activity on occupation, consumption of vegetables and fruits were significantly different among groups classified on fasting plasma glucose (NGT, IFG, and DM). There were also significant differences in age-group, educational level, and history of familial diabetes between these 3 groups. The DM was prevalent in older age-group (29.6%) and the non-literate (22.7%) while IFG was prevalent in participants with elementary school education (17.7%). On the other hand, NGT was prevalent in younger age-groups of 30 to 45 years old.

**Table 1.** Mean, standard deviation, and significance level of demographic and biomedical indicators of sampled population by gender

Variable	Men (N=1,119)	Women (N=1,450)	Significance
Age (years)	41.9±8.3	39.7±8.3	***
Weight (kg)	75.8±11.7	73.2±12.6	***
Height (cm)	169.5±7.6	156.4±8.01	***
BMI (kg/m^2^)	26.5±4.3	29.9±5.4	***
SBP (mmHg)	119.9±14.6	119.4±17.2	ns
DBP (mmHg)	78.4±14.2	77.9±22.9	ns
Daily work (hour/day)	7.4±4.08	5.2±3.2	***
Walking (day/week)	2.6±2.6	1.9±2.4	***
Leisure-time exercise (day/week) Exercise (day/week)	1.4±0.8	1.6±0.7	***
Educational level—No. (%)			
Non-literate	111 (10)	466 (32.1)	
Elementary school	175 (15.6)	577 (39.7)	
Secondary school	175 (15.6)	242 (16.7)	
High school graduate	258 (23.1)	93 (6.5)	***
Tertiary	400 (35.7)	72 (5)	
Family history of diabetes			
Yes	155 (13.9)	258 (17.8)	**
No	964 (86.1)	1,192 (82.2)	

**p<0.01;

***P<0.001; BMI=Body mass index; DBP=Diastolic blood pressure; ns=Non-significant; SBP=Systolic blood pressure

**Table 2.** Distribution of participants at high risk of diabetes according to the criteria of National Diabetes Prevention and Control Committee by gender

Variable	Men (N=1,119)	Women (N=1,450)	Total (N=2,569)
Family history of diabetes	155 (13.9%)	258 (17.8%)	413 (16.1%)
BMI (≥30 kg/m^2^)	217 (19.4%)	710 (48.9%)	927 (36.1%)
Systolic blood pressure (≥140 mmHg)	116 (10.3%)	209 (14.4%)	325 (12.7%)
Diastolic blood pressure (≥90 mmHg)	286 (25.6%)	337 (23.2%)	623 (24.3%)
History of gestational diabetes	-	23 (1.6%)	23 (0. 9%)
History of abortion (≥2 times)	-	120 (8.3%)	120 (4.7%)
History of giving birth to a baby weighing >4 kg	-	288 (19.8%)	288 (11.2%)
History of stillbirth	-	128 (8.8%)	128 (5%)

BMI=Body mass index

Significant differences were observed between NGT and DM (p<0.001) and IFG with DM (p<0.01) in age-groups, NGT and DM (p<0.05) in weight, NGT, and IFG (p<0.05) and IFG with DM (p<0.001) in the consumption of vegetables, and NGT with DM (p<0.05) in consumption of fried food. There were also significant differences between NGT and DM (p<0.01), IFG and DM (p<0.05) in physical activity, NGT with DM (p<0.01), and IFG and DM (p<0.001) in triglyceride. Differences between NGT and DM (p<0.001) and IFG with DM (p<0.001) in cholesterol were also significant.

**Table 3.** Number, percentage, and significance level for gender, age-group, educational level, physical activity level, smoking, and consumption of saturated oil of high- and low-risk populations

Variable	High risk (N=1,336)	Low risk (N=1,233)	Significance
Gender			
Male	382 (34.1)	737 (65.9)	***
Female	954 (65.8)	496 (34.2)	***
Age (completed years)	8.1±40.1	41.1±8.5	***
30-35	415 (47.9)	452 (52.1)	***
36-45	582 (53.5)	505 (46.5)	
46-55	238 (53.7)	205 (46.3)	
56-65	100 (58.5)	71 (41.5)	
Educational Level			
Non-literate	349 (60.8)	225 (39.2)	***
Elementary school	452 (60.0)	301 (40.0)	
Secondary school	219 (52.5)	198 (47.5)	
High school graduate	144 (40.9)	208 (59.1)	
Tertiary	172 (36.4)	301 (63.6)	
Physical activity			
Sedentary	732 (60.7)	473 (39.3)	***
Moderate	517 (46.8)	588 (53.2)	
Heavy	87 (33.6)	172 (66.4)	
Smoking	363 (82.9)	75 (17.1)	***
Consumption of saturated oil	912 (84.6)	166 (15.4)	***

***p<0.001

The odds ratios and their 95% confidence intervals for diabetes (DM) and IFG relative to group with normal glucose level are shown in Table 5. Triglyceride (TG), total cholesterol, family history of diabetes were positively associated with diabetes. Physical activity on occupation, consumption of vegetables and fruits, and younger age had a protective effect whereas IFG was associated with elementary school education.

## DISCUSSION

More than 50% of the surveyed population was found high-risk diabetes. This was expected given that more than one-third of the people (36.3%) were obese, and about one-third were hypertensive. The percentage of women at high risk of diabetes was higher than men. Although pregnancy complications has contributed to the higher percentage of high-risk diabetes in women, frequency of diabetes-associated factors were greater in women than men. Furthermore, average BMI was significantly higher in women than men in contrast to average daily work and walking which was lower in women than men. In our study, majority of women were either non-literate or with elementary school education. Majority of women (89.5%) spent most of their time at home due to lack of opportunity for work outside the house.

High-risk diabetes was associated significantly with increase in age and lower level of education (Table 3).

Health literacy enables individuals to manage their health. Evidence suggests that individuals with the greatest health burdens have least access to simple and understandable health information (22). Sedentary physical activity, smoking, and consumption of saturated oil were associated with high-risk diabetes (Table 3). This finding is consistent with earlier findings that unhealthy lifestyle has association with the higher risk of diabetes, obesity, and hypertension.

**Table 4.** Statistically significant demographic and biomedical indicators affecting individuals classified as NGT, IFG, and DM in high-risk population

Variable	NGT	IFG	DM	Significance
Number (%)	606 (68.8)	118 (13.4)	157 (17.8)	-
Age (years)	41.1±8.3	41.8±8.4	44.7±8.4	***
FBS (mg/dL)	91.3±13.6	115.4±4.4	184.4±74.2	***
TC (mg/dL)	203.6±50.0	212±47.6	236.8±68.4	***
TG (mg/dL)	172.1±96.9	186±1.6	251±153	***
Physical activity on occupation (day/week)	4.5±2.96	4.5±2.9	3.7±3.1	*
Consumption of vegetables and fruits fruits (day/week)	4.4±2.4	3.4±2.5	3.9±3.0	**
Age (completed years)				
30-35	182 (75.8)	35 (14.6)	23 (9.6)	***
36-45	279 (70.6)	48 (12.2)	68 (17.2)	
46-55	104 (62.3)	24 (14.4)	39 (23.4)	
56-65	39 (54.9)	11 (15.5)	21 (29.6)	
Education—No. (%)				
Non-literate	160 (64.8)	31 (12.6)	56 (22.7)	**
Elementary school	203 (65.3)	55 (17.7)	53 (17)	
Secondary school	95 (74.2)	13 (10.2)	20 (15.6)	
High school graduate	67 (76.2)	9 (10.2)	12 (13.6)	
Tertiary	81 (75.7)	10 (9.3)	16 (15)	
Family history of diabetes—No. (%)				
Yes	59 (24.1)	79 (33.2)	107 (43.6)	***
No	459 (72.2)	92 (14.4)	85 (13.4)	

*p<0.05;

**p<0.01;

***p<0.001; BMI=Body mass index; DM=Diabetes mellitus; FBS=Fasting blood sugar; IFG=Impaired fasting glucose; NGT=Normal glucose tolerance; TC=Total cholesterol; TG=Triglyceride

Family history of diabetes, high BMI, and smoking were significant causes of diabetes found in those studies. An inverse relationship was observed between obesity with occupational and leisure-time activities. Consistent with our study, intake of animal fat, sedentary profession, and lack of exercise were associated with risk of diabetes (11-16,23).

Screening of high-risk asymptomatic individuals provided the first representative, population-based estimates of the frequency of unknown diabetes and IFG in the adults of Yasuj. The percentage of participants at high risk of diabetes (17.8%) in this study was higher compared to the findings of the previous studies in Iran and other countries, like Pakistan, Hong Kong, the Netherlands, and Canada, with 10.4%, 14.6%, 5.6%, and 13.2% prevalence respectively (24-27). The high percentage of undiagnosed diabetes found in our study, which was due to low awareness and non-literacy of individuals at high risk of diabetes, may have contributed to higher percentage of at-risk population in our study. On the other hand, the rate of previously-undiagnosed (newly-diagnosed) diabetes was higher than known diabetes in contrast to findings of other surveys conducted in Iran.

The prevalence of diagnosed and undiagnosed diabetes in a large urban Iranian population aged ≥20 years was estimated at 8.1%, 5.1% in men and 10%, 4.7% in women respectively (16). Screening programmes conducted in different parts of Iran revealed that nearly half of the type 2 diabetics were unaware of their problem (15). The prevalence of diabetes in the Korean population was estimated by Kim *et al*. (28) at 7.6%, consisting of 4.4% previously-diagnosed and 3.3% newly-diagnosed individuals, which is comparable with the estimates from Western countries, such as the USA and Australia. Recent Asian studies have estimated diabetes prevalence of 5.5% in China, 9.6% in Thailand, and 9.1% for men and 10.8% for women in rural Japan (29). Overestimation of newly-diagnosed diabetes prevalence in our study might be due to the fact that we have selected the high-risk individuals based on assigned laboratory diagnostics, thus excluding a substantial proportion of people not at high-risk and those who did not participate in screening for diabetes.

**Table 5.** Odds ratio (OR), 95% confidence interval (95% CI), and significance level of IFG and DM for demographic and biomedical indicators

Variable	IFG		DM	
	OR (95% CI)	Significance	OR (95% CI)	Significance
TG (mg/dL)	1.001 (0.999-1.003)	ns	1.004 (1.002-1.006)	***
TC (mg/dL)	1.003 (0.999-1.007)	ns	1.008 (1.004-1.012)	***
Physical activity on occupation (day/week))	0.996 (0.925-1.072)	ns	0.920 (0.858-0.986)	*
Consumption of vegetables and fruits (day/week)	0.923 (0.836-1.019)	ns	1.099 (1.002-1.250)	*
Age (completed years)				
30-35	0.741 (0.283-1.940)	ns	0.245 (0.102-0.591)	**
36-45	0.601 (0.240-1.506)	ns	0.424 (0.196-0.920)	*
46-55	0.788 (0.302-2.058)	ns	0.589 (0.261-1.320)	ns
56-65	-	-	-	-
Education				
Non-literate	1.323 (0.560-3.122)	ns	1.355 (0.634-2.892)	ns
Elementary school	2.257 (1.037-4.909	*	1.724 (0.846-3.513)	ns
Secondary school	1.032 (0.399-2.672)	ns	1.251 (0.528-2.964)	ns
High school graduate	1.155 (0.726-3.134)	ns	1.214 (0.497-2.969)	ns
Tertiary	-	-	-	-
Family history of diabetes				
Yes	1.557 (0.970-2.490)	ns	3.129 (2.036-4.810)	***
No	-	-	-	-

*p<0.05;

**p<0.01;

***p<0.001; ns=Non-significant; TC=Total cholesterol; TG=Triglyceride

In agreement with other studies (28-32), this study confirms that established risk factors, such as ageing, dyslipidaemia, low physical activity, non-literacy, and a family history of diabetes are associated with diabetes. A Korean study found that prevalence of diabetes increases with age and peaks in the oldest age-group. Diabetes was also found to be associated with ageing, triglyceride, HDL cholesterol, educational levels, alcohol consumption, exercise, and family history of diabetes (28). Using multiple logistic regressions, an epidemiologic study of diabetes in Turkey found age, familial diabetes, and education to be associated with diabetes in men but was protective for diabetes and IGT in women (29). Azimi-Nezhad *et al*. (2007) reported diabetes mellitus to be most prevalent among the older age-group comprising retired and non-literate individuals (30). Age, sex, hypertension, family history of diabetes, and triglyceride were independently associated with diabetes in similar studies (31,32).

Findings suggest that dietary and behavioural habits of IFG and DM participants were less favourable than normal population. We found that higher intake of dietary fibre, such as fruits and vegetables, has protective effect on diabetes. Even though anecdotal evidences suggest that fruits, vegetables, low-fat dairy, unsaturated fat, and fibre reduce the risk of diabetes, it is not scientifically proven yet (33).

Previous studies have established the associations between hypertension, overweight, and obesity with higher rates of diabetes (34,35) in contrast to our study where non-significant differences in BMI were observed between normal, IFG and diabetes groups. This is due to the fact that the population which was screened for high-risk diabetes included obesity (BMI ≥30) and hypertension as criteria for the study. In addition, waist-circumference, which indicates an android type of fat deposition and abdominal obesity and appears to be a major independent risk factor of both diabetes and pre-diabetes of participants (36), was not measured in our study.

Interestingly, in contrast to our expectation, we did not find a statistically significant difference between the prevalence of diabetes in men and women despite higher contribution of women to high-risk population. Consistent with our results, Al-Lawati *et al*. (37) in Oman and Riste *et al*. (38) in Britain did not find any significant difference between the prevalence of diabetes in men and women in diverse ethnic groups. A recent survey in Uzbekistan found a slightly higher prevalence of diabetes in men, although impaired glucose tolerance (IGT) was more common among women (39). Although the rate of diabetes among women in Pakistan was not higher than in men, the rate of impaired glucose tolerance has been reported to be significantly higher among females compared to males (24). In earlier studies in Iran and some Arab countries, both diabetes and IFG have been reported more prevalent among women than men (14-18). These discrepancies may be due to differential distribution in risk factors between men and women.

In our study, households for both questionnaire and physical examinations were recruited through door-to door visits, and 97.3% of those who answered at the door agreed to join the study. However, the rate of cooperation of high-risk individuals for laboratory studies was 66.9% which is lower than the participation rate in the field survey due to individual self-referral to the laboratory. The lack of full participation of high-risk population in laboratory testing was the limitation of our study.

### Conclusions

The study suggests that diabetes and factors associated with its occurrence are common health problems in this region. The high prevalence of DM and considerable percentage of newly-diagnosed diabetes signifies the role of screening programmes. While it is generally agreed that available data do not support universal diabetes screening, some recent reports suggest that screening programmes targeting individuals with multiple diabetes risk factors may be worthwhile (40). With respect to long-term complications, early identification would shift the focus of diabetes care towards a more preventive one. Primary prevention through lifestyle modifications may have a critical role in the control of diabetes. The results underline the need to increase public awareness and to emphasize the value of lifestyle modification towards healthy nutrition and increased physical activity.
